# *catena*-Poly[[(1,10-phenanthroline-κ^2^ *N*,*N*′)cadmium(II)]-μ-oxalato-κ^4^ *O* ^1^,*O* ^2^:*O* ^1′^,*O* ^2′^]

**DOI:** 10.1107/S1600536810040341

**Published:** 2010-10-23

**Authors:** Yao-Kang Lv, Li-Hua Gan, Yong-Jie Cao, Biao-Feng Gao, Liu-Hua Chen

**Affiliations:** a Chemistry Department, Tongji University, Shanghai 200092, People’s Republic of China

## Abstract

In the title complex, [Cd(C_2_O_4_)(C_12_H_8_N_2_)]_*n*_, the Cd^II^ atom has a distorted octa­hedral coordination, defined by four O atoms from two symmetry-related oxalate ligands and by two N atoms from a bidentate 1,10-phenanthroline ligand. Each oxalate ligand bridges two Cd^II^ atoms, generating a zigzag chain structure propagating along [100]. The packing of the structure is consolidated by non-classical C—H⋯O hydrogen-bonding inter­actions.

## Related literature

For general background to the rational design and synthesis of metal-organic polymers, see: Kondrashev *et al.* (1985 ▶); Orioli *et al.* (2002 ▶); Athar *et al.* (2008 ▶); Lv *et al.* (2010 ▶). Wu *et al.* (2003 ▶). For related structures, see: Cao *et al.* (2009 ▶); Jeanneau *et al.* (2001 ▶).
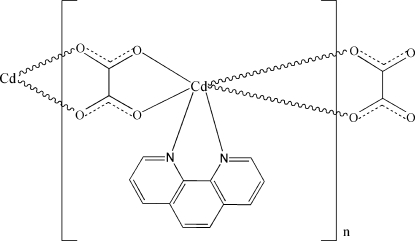


## Experimental

### 

#### Crystal data

[Cd(C_2_O_4_)(C_12_H_8_N_2_)]
*M* *_r_* = 380.62
Orthorhombic, 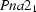

*a* = 9.7199 (2) Å
*b* = 10.3338 (2) Å
*c* = 13.1638 (2) Å
*V* = 1322.22 (4) Å^3^
*Z* = 4
Mo *K*α radiation
μ = 1.67 mm^−1^
*T* = 296 K
0.29 × 0.14 × 0.10 mm

#### Data collection

Bruker APEXII area-detector diffractometer
Absorption correction: multi-scan (*SADABS*; Sheldrick, 1996 ▶) *T*_min_ = 0.76, *T*_max_ = 0.85
11056 measured reflections
2892 independent reflections
2386 reflections with *I* > 2σ(*I*)
*R*_int_ = 0.034

#### Refinement

*R*[*F* ^2^ > 2σ(*F* ^2^)] = 0.027
*wR*(*F* ^2^) = 0.066
*S* = 1.00
2892 reflections
191 parameters
1 restraint
H-atom parameters constrained
Δρ_max_ = 0.37 e Å^−3^
Δρ_min_ = −0.27 e Å^−3^
Absolute structure: Flack (1983 ▶), 1310 Friedel pairs
Flack parameter: 0.33 (4)

### 

Data collection: *APEX2* (Bruker, 2006 ▶); cell refinement: *SAINT* (Bruker, 2006 ▶); data reduction: *SAINT*; program(s) used to solve structure: *SHELXS97* (Sheldrick, 2008 ▶); program(s) used to refine structure: *SHELXL97* (Sheldrick, 2008 ▶); molecular graphics: *DIAMOND* (Brandenburg & Putz, 2004 ▶); software used to prepare material for publication: *SHELXTL* (Sheldrick, 2008 ▶).

## Supplementary Material

Crystal structure: contains datablocks global, I. DOI: 10.1107/S1600536810040341/wm2410sup1.cif

Structure factors: contains datablocks I. DOI: 10.1107/S1600536810040341/wm2410Isup2.hkl

Additional supplementary materials:  crystallographic information; 3D view; checkCIF report

## Figures and Tables

**Table 1:** Selected bond lengths (Å)

Cd1—O1	2.258 (3)
Cd1—O4^i^	2.269 (3)
Cd1—O2	2.271 (3)
Cd1—O3^i^	2.294 (3)
Cd1—N2	2.307 (3)
Cd1—N1	2.338 (3)

Symmetry code: (i) 
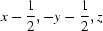
.

**Table 2:** Hydrogen-bond geometry (Å, °)

*D*—H⋯*A*	*D*—H	H⋯*A*	*D*⋯*A*	*D*—H⋯*A*
C4—H4*A*⋯O2^ii^	0.93	2.38	3.106 (6)	134
C7—H7*A*⋯O1^iii^	0.93	2.57	3.289 (6)	134
C11—H11*A*⋯O3^iv^	0.93	2.42	3.302 (7)	159

Symmetry codes: (ii) 
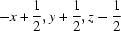
; (iii) 
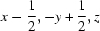
; (iv) 
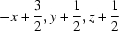
.

**Fig. 1.**
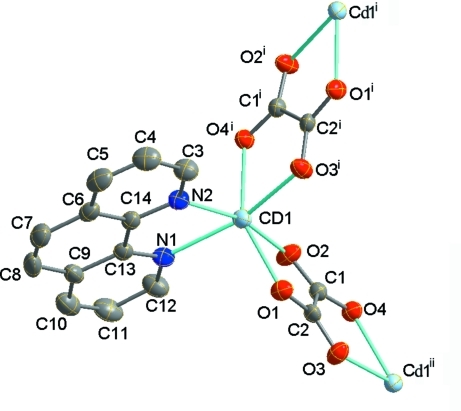
The asymmetric unit and some symmetry-related atoms of the title complex. Displacement ellipsoids are plotted at the 30% probability level and H atoms are not labeled. [Symmetry codes: (i) x-0.5, -y-0.5, z; (ii) x+0.5, -y-0.5, z.]

**Fig. 2.**
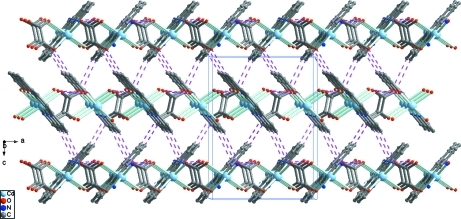
Packing diagram of the title compound viewed down [010]; all H atoms were omitted for clarity.

## supplementary crystallographic information

### Comment

Rational design and synthesis of metal-organic polymers is of current interest in the field of supramolecular chemistry and crystal engineering (Athar *et al.*, 2008; Lv *et al.*, 2010; Wu *et al.*, 2003). Among the anions involved in the formation of such solids, the oxalate anion, which possesses four donor O atoms, plays an important role. Indeed, it can act either as a monodentate or a bidentate chelating ligand and can thus bridge two or more metal atoms in a variety of arrangements, as recently shown with a number of compounds (Kondrashev *et al.*, 1985; Jeanneau *et al.*, 2001; Cao *et al.*, 2009; Orioli *et al.*, 2002). We report here on the synthesis and structure of the title compound, [Cd(C_2_O_4_)(C_12_H_8_N_2_)].

As shown in Fig. 1, the central Cd(II) atom is six-coordinated by four O atoms from two symmetry-related oxalate ligands and two N atoms from a bidendate 1,10-phenanthroline ligand (Table 1), forming a distorted octahedral geometry. Each oxalate ligand bridges two cadmium(II) atoms generating a zigzag chain structure propagating along [100]. Furthermore, there are non-classical C—H···O hydrogen bonds present (Table 2). Together with van der Waals forces they interconnect the zigzag chains and construct a supramolecular network (Fig. 2).

### Experimental

The synthesis of the title complex (I) was carried out by hydrothermal reaction. A mixture of Cd(NO_3_)_2_^.^4H_2_O (1.0 mmol), K_2_C_2_O_4_ (1.0 mmol), 1,10-phenanthroline (1.0 mmol) in 20 ml water was placed in a Teflon-lined stainless steel autoclave and heated at 433 K for 72 h, and then cooled to room temperature over 3 days. The resulting colorless crystals suitable for X-ray analysis were obtained in about 36% yield.

### Refinement

The H atoms bonded to C atoms were positioned geometrically [C—H 0.93 Å *U*_iso_(H) = 1.2*U*_eq_(C)]. The crystal measured was an inversion twin with a 2:1 ratio for the twin domains.

### Crystal data

**Table:**

[Cd(C_2_O_4_)(C_12_H_8_N_2_)]	*F*(000) = 744
*M**_r_* = 380.62	*D*_x_ = 1.912 Mg m^−^^3^
Orthorhombic, *P**n**a*2_1_	Mo *K*α radiation, λ = 0.71073 Å
Hall symbol: P 2c -2n	Cell parameters from 2979 reflections
*a* = 9.7199 (2) Å	θ = 2.5–27.4°
*b* = 10.3338 (2) Å	µ = 1.67 mm^−^^1^
*c* = 13.1638 (2) Å	*T* = 296 K
*V* = 1322.22 (4) Å^3^	Block, colourless
*Z* = 4	0.29 × 0.14 × 0.10 mm

### Data collection

**Table:**

Bruker APEXII area-detector diffractometer	2892 independent reflections
Radiation source: fine-focus sealed tube	2386 reflections with *I* > 2σ(*I*)
graphite	*R*_int_ = 0.034
ω scans	θ_max_ = 27.4°, θ_min_ = 2.5°
Absorption correction: multi-scan (*SADABS*; Sheldrick, 1996)	*h* = −12→12
*T*_min_ = 0.76, *T*_max_ = 0.85	*k* = −13→13
11056 measured reflections	*l* = −17→17

### Refinement

**Table:**

Refinement on *F*^2^	Secondary atom site location: difference Fourier map
Least-squares matrix: full	Hydrogen site location: inferred from neighbouring sites
*R*[*F*^2^ > 2σ(*F*^2^)] = 0.027	H-atom parameters constrained
*wR*(*F*^2^) = 0.066	*w* = 1/[σ^2^(*F*_o_^2^) + (0.0359*P*)^2^] where *P* = (*F*_o_^2^ + 2*F*_c_^2^)/3
*S* = 1.00	(Δ/σ)_max_ = 0.001
2892 reflections	Δρ_max_ = 0.37 e Å^−^^3^
191 parameters	Δρ_min_ = −0.27 e Å^−^^3^
1 restraint	Absolute structure: Flack (1983), 1310 Friedel pairs
Primary atom site location: structure-invariant direct methods	Flack parameter: 0.33 (4)

### Special details

**Table:**

Geometry. All e.s.d.'s (except the e.s.d. in the dihedral angle between two l.s. planes) are estimated using the full covariance matrix. The cell e.s.d.'s are taken into account individually in the estimation of e.s.d.'s in distances, angles and torsion angles; correlations between e.s.d.'s in cell parameters are only used when they are defined by crystal symmetry. An approximate (isotropic) treatment of cell e.s.d.'s is used for estimating e.s.d.'s involving l.s. planes.
Refinement. Refinement of *F*^2^ against ALL reflections. The weighted *R*-factor *wR* and goodness of fit *S* are based on *F*^2^, conventional *R*-factors *R* are based on *F*, with *F* set to zero for negative *F*^2^. The threshold expression of *F*^2^ > σ(*F*^2^) is used only for calculating *R*-factors(gt) *etc*. and is not relevant to the choice of reflections for refinement. *R*-factors based on *F*^2^ are statistically about twice as large as those based on *F*, and *R*- factors based on ALL data will be even larger.

### Fractional atomic coordinates and isotropic or equivalent isotropic displacement parameters (Å^2^)

**Table:**

	*x*	*y*	*z*	*U*_iso_*/*U*_eq_	
Cd1	0.38159 (2)	−0.09300 (2)	0.33607 (7)	0.04611 (10)	
O1	0.5669 (3)	−0.1452 (3)	0.2410 (2)	0.0499 (7)	
O2	0.5193 (3)	−0.2166 (3)	0.4361 (2)	0.0579 (8)	
O3	0.7580 (3)	−0.2606 (3)	0.2421 (2)	0.0558 (8)	
O4	0.6953 (3)	−0.3510 (3)	0.4301 (2)	0.0475 (7)	
N1	0.4604 (4)	0.1011 (3)	0.4063 (3)	0.0488 (9)	
N2	0.2834 (4)	0.0751 (3)	0.2463 (3)	0.0480 (8)	
C1	0.6195 (3)	−0.2664 (4)	0.3923 (4)	0.0395 (11)	
C2	0.6499 (5)	−0.2203 (4)	0.2815 (4)	0.0413 (11)	
C3	0.1925 (5)	0.0621 (5)	0.1726 (3)	0.0591 (12)	
H3A	0.1661	−0.0210	0.1539	0.071*	
C4	0.1343 (5)	0.1662 (7)	0.1214 (4)	0.0689 (15)	
H4A	0.0695	0.1532	0.0704	0.083*	
C5	0.1737 (6)	0.2856 (6)	0.1474 (4)	0.0677 (15)	
H5A	0.1375	0.3563	0.1128	0.081*	
C6	0.2686 (4)	0.3061 (4)	0.2260 (4)	0.0537 (11)	
C7	0.3150 (6)	0.4299 (5)	0.2568 (5)	0.0691 (14)	
H7A	0.2827	0.5028	0.2231	0.083*	
C8	0.4037 (5)	0.4444 (4)	0.3329 (9)	0.0735 (13)	
H8A	0.4315	0.5273	0.3512	0.088*	
C9	0.4585 (5)	0.3335 (4)	0.3883 (4)	0.0566 (11)	
C10	0.5520 (6)	0.3424 (6)	0.4680 (4)	0.0738 (15)	
H10A	0.5841	0.4231	0.4886	0.089*	
C11	0.5964 (6)	0.2351 (7)	0.5155 (5)	0.0736 (18)	
H11A	0.6584	0.2408	0.5691	0.088*	
C12	0.5478 (6)	0.1150 (5)	0.4831 (4)	0.0666 (14)	
H12A	0.5780	0.0412	0.5169	0.080*	
C13	0.4163 (4)	0.2091 (4)	0.3576 (3)	0.0436 (11)	
C14	0.3214 (4)	0.1959 (4)	0.2760 (3)	0.0424 (9)	

### Atomic displacement parameters (Å^2^)

**Table:**

	*U*^11^	*U*^22^	*U*^33^	*U*^12^	*U*^13^	*U*^23^
Cd1	0.04105 (14)	0.04202 (14)	0.05528 (16)	0.00004 (11)	−0.0025 (2)	0.0066 (2)
O1	0.0474 (16)	0.0561 (17)	0.0462 (17)	0.0046 (15)	0.0020 (14)	0.0163 (16)
O2	0.0529 (18)	0.070 (2)	0.0505 (17)	0.0190 (16)	0.0150 (16)	0.0229 (17)
O3	0.0573 (19)	0.0592 (17)	0.0508 (17)	0.0164 (15)	0.0141 (15)	0.0105 (15)
O4	0.0469 (15)	0.0471 (15)	0.0484 (16)	0.0047 (13)	0.0033 (13)	0.0097 (14)
N1	0.0444 (19)	0.056 (2)	0.0457 (19)	−0.0024 (16)	−0.0074 (15)	0.0050 (18)
N2	0.0466 (19)	0.051 (2)	0.0460 (19)	0.0014 (15)	−0.0064 (16)	0.0079 (17)
C1	0.042 (3)	0.038 (2)	0.039 (2)	−0.0019 (18)	−0.0002 (18)	0.001 (2)
C2	0.044 (2)	0.032 (2)	0.049 (3)	−0.0045 (19)	0.006 (2)	0.006 (2)
C3	0.057 (3)	0.069 (3)	0.051 (3)	−0.002 (2)	−0.011 (2)	0.006 (2)
C4	0.063 (3)	0.089 (4)	0.054 (3)	0.001 (3)	−0.013 (2)	0.018 (3)
C5	0.061 (3)	0.074 (4)	0.068 (4)	0.020 (3)	0.002 (3)	0.031 (3)
C6	0.047 (2)	0.050 (2)	0.064 (3)	0.0105 (19)	0.013 (2)	0.015 (2)
C7	0.070 (3)	0.049 (3)	0.088 (4)	0.013 (2)	0.009 (3)	0.010 (3)
C8	0.082 (3)	0.0364 (19)	0.102 (4)	−0.0012 (19)	0.031 (5)	−0.002 (5)
C9	0.051 (3)	0.049 (3)	0.070 (3)	−0.013 (2)	0.013 (2)	−0.010 (2)
C10	0.074 (4)	0.074 (4)	0.074 (4)	−0.020 (3)	0.012 (3)	−0.015 (3)
C11	0.072 (4)	0.100 (5)	0.049 (3)	−0.026 (3)	−0.011 (3)	−0.008 (3)
C12	0.066 (3)	0.074 (3)	0.060 (3)	−0.014 (3)	−0.018 (3)	0.012 (3)
C13	0.0400 (18)	0.045 (2)	0.046 (3)	−0.0005 (15)	0.0091 (18)	0.004 (2)
C14	0.043 (2)	0.041 (2)	0.043 (2)	0.0026 (17)	0.0074 (18)	0.0054 (18)

### Geometric parameters (Å, °)

**Table:**

Cd1—O1	2.258 (3)	C4—C5	1.336 (9)
Cd1—O4^i^	2.269 (3)	C4—H4A	0.9300
Cd1—O2	2.271 (3)	C5—C6	1.402 (7)
Cd1—O3^i^	2.294 (3)	C5—H5A	0.9300
Cd1—N2	2.307 (3)	C6—C14	1.411 (5)
Cd1—N1	2.338 (3)	C6—C7	1.416 (7)
O1—C2	1.240 (5)	C7—C8	1.330 (11)
O2—C1	1.242 (5)	C7—H7A	0.9300
O3—C2	1.244 (5)	C8—C9	1.459 (9)
O3—Cd1^ii^	2.294 (3)	C8—H8A	0.9300
O4—C1	1.247 (5)	C9—C10	1.392 (7)
O4—Cd1^ii^	2.269 (3)	C9—C13	1.409 (6)
N1—C12	1.329 (6)	C10—C11	1.344 (9)
N1—C13	1.356 (5)	C10—H10A	0.9300
N2—C3	1.319 (6)	C11—C12	1.394 (8)
N2—C14	1.359 (5)	C11—H11A	0.9300
C1—C2	1.563 (5)	C12—H12A	0.9300
C3—C4	1.391 (7)	C13—C14	1.422 (6)
C3—H3A	0.9300		
			
O1—Cd1—O4^i^	151.38 (10)	C5—C4—C3	118.3 (5)
O1—Cd1—O2	73.54 (9)	C5—C4—H4A	120.9
O4^i^—Cd1—O2	90.60 (10)	C3—C4—H4A	120.9
O1—Cd1—O3^i^	87.77 (11)	C4—C5—C6	121.1 (5)
O4^i^—Cd1—O3^i^	73.03 (10)	C4—C5—H5A	119.5
O2—Cd1—O3^i^	104.50 (12)	C6—C5—H5A	119.5
O1—Cd1—N2	103.06 (12)	C5—C6—C14	117.5 (4)
O4^i^—Cd1—N2	98.14 (12)	C5—C6—C7	123.9 (5)
O2—Cd1—N2	164.61 (13)	C14—C6—C7	118.6 (5)
O3^i^—Cd1—N2	90.22 (13)	C8—C7—C6	121.6 (5)
O1—Cd1—N1	99.37 (13)	C8—C7—H7A	119.2
O4^i^—Cd1—N1	105.35 (12)	C6—C7—H7A	119.2
O2—Cd1—N1	93.45 (13)	C7—C8—C9	121.7 (5)
O3^i^—Cd1—N1	161.94 (12)	C7—C8—H8A	119.2
N2—Cd1—N1	72.07 (12)	C9—C8—H8A	119.2
C2—O1—Cd1	115.5 (3)	C10—C9—C13	117.9 (5)
C1—O2—Cd1	115.2 (3)	C10—C9—C8	124.3 (5)
C2—O3—Cd1^ii^	116.0 (3)	C13—C9—C8	117.8 (5)
C1—O4—Cd1^ii^	115.6 (3)	C11—C10—C9	120.4 (5)
C12—N1—C13	118.3 (4)	C11—C10—H10A	119.8
C12—N1—Cd1	127.1 (3)	C9—C10—H10A	119.8
C13—N1—Cd1	114.5 (3)	C10—C11—C12	119.0 (5)
C3—N2—C14	119.1 (4)	C10—C11—H11A	120.5
C3—N2—Cd1	125.3 (3)	C12—C11—H11A	120.5
C14—N2—Cd1	115.6 (3)	N1—C12—C11	123.0 (5)
O2—C1—O4	124.6 (5)	N1—C12—H12A	118.5
O2—C1—C2	117.1 (4)	C11—C12—H12A	118.5
O4—C1—C2	118.3 (4)	N1—C13—C9	121.5 (4)
O1—C2—O3	125.5 (5)	N1—C13—C14	118.9 (4)
O1—C2—C1	117.9 (4)	C9—C13—C14	119.5 (4)
O3—C2—C1	116.6 (4)	N2—C14—C6	120.5 (4)
N2—C3—C4	123.4 (5)	N2—C14—C13	118.7 (3)
N2—C3—H3A	118.3	C6—C14—C13	120.7 (4)
C4—C3—H3A	118.3		
			
O4^i^—Cd1—O1—C2	−54.2 (5)	O2—C1—C2—O3	172.9 (5)
O2—Cd1—O1—C2	4.5 (3)	O4—C1—C2—O3	−8.0 (5)
O3^i^—Cd1—O1—C2	−101.3 (4)	C14—N2—C3—C4	1.2 (7)
N2—Cd1—O1—C2	169.0 (3)	Cd1—N2—C3—C4	179.0 (4)
N1—Cd1—O1—C2	95.4 (3)	N2—C3—C4—C5	0.9 (8)
O1—Cd1—O2—C1	−7.7 (3)	C3—C4—C5—C6	−1.6 (8)
O4^i^—Cd1—O2—C1	148.1 (3)	C4—C5—C6—C14	0.4 (7)
O3^i^—Cd1—O2—C1	75.5 (3)	C4—C5—C6—C7	179.7 (5)
N2—Cd1—O2—C1	−87.0 (6)	C5—C6—C7—C8	179.0 (6)
N1—Cd1—O2—C1	−106.5 (3)	C14—C6—C7—C8	−1.8 (8)
O1—Cd1—N1—C12	−78.2 (4)	C6—C7—C8—C9	0.3 (10)
O4^i^—Cd1—N1—C12	87.2 (4)	C7—C8—C9—C10	179.6 (6)
O2—Cd1—N1—C12	−4.4 (4)	C7—C8—C9—C13	1.1 (9)
O3^i^—Cd1—N1—C12	169.5 (4)	C13—C9—C10—C11	−2.0 (7)
N2—Cd1—N1—C12	−179.0 (4)	C8—C9—C10—C11	179.4 (6)
O1—Cd1—N1—C13	97.6 (3)	C9—C10—C11—C12	0.4 (9)
O4^i^—Cd1—N1—C13	−96.9 (3)	C13—N1—C12—C11	−0.3 (8)
O2—Cd1—N1—C13	171.5 (3)	Cd1—N1—C12—C11	175.5 (4)
O3^i^—Cd1—N1—C13	−14.6 (6)	C10—C11—C12—N1	0.8 (9)
N2—Cd1—N1—C13	−3.2 (3)	C12—N1—C13—C9	−1.5 (6)
O1—Cd1—N2—C3	88.0 (4)	Cd1—N1—C13—C9	−177.7 (3)
O4^i^—Cd1—N2—C3	−72.7 (4)	C12—N1—C13—C14	−179.2 (4)
O2—Cd1—N2—C3	163.3 (4)	Cd1—N1—C13—C14	4.5 (5)
O3^i^—Cd1—N2—C3	0.2 (4)	C10—C9—C13—N1	2.6 (6)
N1—Cd1—N2—C3	−176.3 (4)	C8—C9—C13—N1	−178.7 (5)
O1—Cd1—N2—C14	−94.2 (3)	C10—C9—C13—C14	−179.6 (4)
O4^i^—Cd1—N2—C14	105.1 (3)	C8—C9—C13—C14	−1.0 (6)
O2—Cd1—N2—C14	−18.9 (6)	C3—N2—C14—C6	−2.5 (6)
O3^i^—Cd1—N2—C14	178.0 (3)	Cd1—N2—C14—C6	179.5 (3)
N1—Cd1—N2—C14	1.6 (3)	C3—N2—C14—C13	178.1 (4)
Cd1—O2—C1—O4	−169.5 (3)	Cd1—N2—C14—C13	0.1 (5)
Cd1—O2—C1—C2	9.5 (4)	C5—C6—C14—N2	1.7 (6)
Cd1^ii^—O4—C1—O2	−174.2 (3)	C7—C6—C14—N2	−177.6 (4)
Cd1^ii^—O4—C1—C2	6.8 (4)	C5—C6—C14—C13	−178.9 (4)
Cd1—O1—C2—O3	−179.9 (4)	C7—C6—C14—C13	1.8 (6)
Cd1—O1—C2—C1	−1.5 (5)	N1—C13—C14—N2	−3.2 (6)
Cd1^ii^—O3—C2—O1	−176.9 (4)	C9—C13—C14—N2	179.0 (4)
Cd1^ii^—O3—C2—C1	4.6 (4)	N1—C13—C14—C6	177.4 (4)
O2—C1—C2—O1	−5.6 (5)	C9—C13—C14—C6	−0.4 (6)
O4—C1—C2—O1	173.4 (5)		

Symmetry codes: (i) *x*−1/2, −*y*−1/2, *z*; (ii) *x*+1/2, −*y*−1/2, *z*.

### Hydrogen-bond geometry (Å, °)

**Table:**

*D*—H···*A*	*D*—H	H···*A*	*D*···*A*	*D*—H···*A*
C4—H4A···O2^iii^	0.93	2.38	3.106 (6)	134
C7—H7A···O1^iv^	0.93	2.57	3.289 (6)	134
C11—H11A···O3^v^	0.93	2.42	3.302 (7)	159

Symmetry codes: (iii) −*x*+1/2, *y*+1/2, *z*−1/2; (iv) *x*−1/2, −*y*+1/2, *z*; (v) −*x*+3/2, *y*+1/2, *z*+1/2.
